# The role of cholesterol metabolism in tumor therapy, from bench to bed

**DOI:** 10.3389/fphar.2023.928821

**Published:** 2023-04-06

**Authors:** Wenhao Xia, Hao Wang, Xiaozhu Zhou, Yan Wang, Lixiang Xue, Baoshan Cao, Jiagui Song

**Affiliations:** ^1^ Cancer Center of Peking University Third Hospital, Beijing, China; ^2^ School of Basic Medical Sciences, Peking University Health Science Center, Beijing, China; ^3^ Department of Radiation Oncology, Peking University Third Hospital, Beijing, China; ^4^ Department of Clinical Pharmacy, School of Pharmacy, Capital Medical University, Beijing, China; ^5^ Third Hospital Institute of Medical Innovation and Research, Beijing, China; ^6^ Department of Medical Oncology and Radiation Sickness, Peking University Third Hospital, Beijing, China; ^7^ State Key Laboratory of Natural and Biomimetic Drugs, Peking University as the Third Responsibility Unit of Song Jiagui, Beijing, China

**Keywords:** cholesterol, cholesterol metabolism, tumor therapy, pharmacological targets, clinical trial

## Abstract

Cholesterol and its metabolites have important biological functions. Cholesterol is able to maintain the physical properties of cell membrane, play an important role in cellular signaling, and cellular cholesterol levels reflect the dynamic balance between biosynthesis, uptake, efflux and esterification. Cholesterol metabolism participates in bile acid production and steroid hormone biosynthesis. Increasing evidence suggests a strict link between cholesterol homeostasis and tumors. Cholesterol metabolism in tumor cells is reprogrammed to differ significantly from normal cells, and disturbances of cholesterol balance also induce tumorigenesis and progression. Preclinical and clinical studies have shown that controlling cholesterol metabolism suppresses tumor growth, suggesting that targeting cholesterol metabolism may provide new possibilities for tumor therapy. In this review, we summarized the metabolic pathways of cholesterol in normal and tumor cells and reviewed the pre-clinical and clinical progression of novel tumor therapeutic strategy with the drugs targeting different stages of cholesterol metabolism from bench to bedside.

## 1 Introduction

Cholesterol is a ubiquitous sterol present in vertebrates with multiple biological functions. Cholesterol is an essential lipid component of the mammalian cell membrane that can maintain membrane integrity and mobility and form membrane microstructures (Cerqueira et al., 2016). In addition to serving as a membrane structural and functional component, cholesterol produces various oxysterol through enzymatic and non-enzymatic pathways. Cholesterol also represents a precursor of bile acid, and its oxidative effect allows for the biosynthesis of steroid hormones in the steroid-producing tissues (Luu et al., 2016). Cholesterol metabolism homeostasis is maintained by a complex network that regulates cholesterol biosynthesis, uptake, efflux, and storage (Giacomini et al., 2021). In addition, cholesterol also interacts with a variety of proteins, including receptors, channels and enzymes, which are thought to regulate protein stability, localization and activity (Hulce et al., 2013).

Tumor cells are highly proliferative and therefore rely on cholesterol to meet substantially increased nutrient needs for membrane synthesis and support their uncontrolled growth, thereby promoting tumorigenesis and progression (Riscal et al., 2019). Indeed, cholesterol, cholesterol derivatives and cholesterol synthesis intermediates can regulate tumor cell proliferation, motility, stemness and drug resistance (Kopecka et al., 2020a). Given these important functions of cholesterol metabolism in cancer, drugs targeting cholesterol metabolism and tumor treatment strategies have become a hot topic in the field of tumor research and have made significant progress in recent years. In this review, we introduce the metabolic pathways of cholesterol in normal and cancer cells, its role in the tumor therapy, and the latest progress in therapeutic drugs targeting different stages of cholesterol metabolism.

## 2 Overview of the cholesterol metabolism in normal cells

Cholesterol metabolism including biosynthesis, uptake, efflux and storage is a complex and important process under normal physiological conditions. In brief, cholesterol biosynthesis starts with acetyl-coA and involves synergy of more than 20 enzymes, most of them on the membrane of the endoplasmic reticulum (ER) (Luo et al., 2020). Several steps are tightly regulated throughout the process, and some intermediates produced during the process can be transferred and used as precursors for the biosynthesis of other compounds (Cerqueira et al., 2016; Luo et al., 2020). The biosynthesis cascade of cholesterol occurs in almost every mammalian cell, especially liver synthesis accounts for about 50% of the total cholesterol biosynthesis (Luo et al., 2020).

Cholesterol uptake consists of NPC1L1 (Niemann–Pick C1-like-1) protein-mediated absorption from the intestinal lumen and LDLR-mediated subsequent absorption from the blood (Luo et al., 2020). NPC1L1 is a glycosylated, multi-spanning membrane protein specifically expressed on the apical surface of enterocytes and the membrane of bile canaliculi of human hepatocytes (Altmann et al., 2004). It is a key mediator of cholesterol uptake and controls cholesterol uptake in enterocytes through clathrin-mediated endocytosis (Luo et al., 2020). The human *NPC1L1* gene is activated by SREBP2 and is upregulated by hepatocyte nuclear factor 4α (HNF4α) (Iwayanagi et al., 2008).

Although almost all mammalian cells can produce cholesterol, only hepatocytes, adrenal cells, and gonad cells are able to catabolize cholesterol. Thus, excess cholesterol of peripheral tissues is converted to cholesterol esters stored in lipid droplets or moved to the liver that can be converted to bile acids and excreted into the digestive system (Ouimet et al., 2019). Mechanistically, four members of the ATP binding cassette (ABC) transporter superfamily: ABC subfamily A member 1 (ABCA1), ABC subfamily G (ABCG) members 1, 5, and 8 regulate cholesterol efflux in a cell-type-specific manner. ABCA1 is widely expressed throughout the body and its main receptor mediating cholesterol efflux is lipid-free apolipoprotein A-I (apoA-I) (Rosenson et al., 2012) and produces HDL particles. ABCG1 is most abundant in macrophages, lower in hepatocytes, and absent in enterocytes (Kennedy et al., 2005). However, ABCG5 and ABCG8 are nearly exclusively expressed at the apical surface of enterocytes and hepatocytes, forming a heterodimer mediating the excretion of cholesterol into the bile and intestinal lumen (Graf et al., 2003).

As mentioned above, excess intracellular cholesterol is usually converted to cholesterol esters, which is an important means to prevent free cholesterol accumulation in cells. The formation of cholesterol esters is mediated by acyl coenzyme A cholesterol acetyltransferase (ACAT) (Petan et al., 2018). To date, two ACAT isoenzymes have been reported in mammals, including ACAT1 and ACAT2. ACAT1 is widely expressed throughout the body and is most abundant in macrophages, epithelial cells and steroid hormone-producing cells, indicating its involvement in maintaining cholesterol homeostasis, while ACAT2 is mainly expressed in enterocytes and also in hepatocytes, suggesting that it contributes to lipoprotein biosynthesis and assembly (Luo et al., 2020).

The molecular mechanism of cholesterol metabolism is strictly regulated to maintain cholesterol homeostasis, not only satisfy cell growth and proliferation with enough cholesterol, but also avoid excessive cholesterol accumulation. Cholesterol homeostasis is mainly regulated by 2 families of transcription factors: the sterol regulatory element binding proteins (SREBPs) and the liver X receptors (LXRs) (Luo et al., 2020). SREBP1 mainly regulates the genes involved in fatty acid (FA) synthesis, while SREBP2 controls the gene of the cholesterol biosynthesis pathway (Horton et al., 2003). When the cholesterol content is present in endoplasmic reticulum (ER) is low, SREBP2 activates the transcription and expression of the cholesterol biosynthetic enzymes HMGCR, increases the expression of the *NPC1L1* and *LDLR* genes (Luo et al., 2020) to increase the *de novo* cholesterol synthesis (Nohturfft and Zhang, 2009; Cai et al., 2019). When cholesterol content in endoplasmic reticulum (ER) is high, the activation of SREBP2 and cholesterol synthesis are blocked. Moreover, LXRs promotes activation of genes associated with bile acid generation (CYP7A1), cholesterol excretion (ABCG5, ABCG8), and reverse cholesterol transport (ABCA1, ABCG1) (Giacomini et al., 2021), ultimately promoting the elimination of the excess of cellular cholesterol.

Although cholesterol is essential for membrane fluidity and structural maintenance, signaling regulation, and energy storage, most mammalian cells cannot directly process cholesterol through the catalytic reaction, but may modify their steroid skeleton, which further generate oxysterols eventually and bile acid *via* cholesterol efflux ultimately upon the content of cholesterol is overload (Luu et al., 2016; Riscal et al., 2019). Oxysterols are oxidized forms of cholesterol, which present at extremely low concentrations in human (van Reyk et al., 2006). Oxysterols regulate cellular cholesterol homeostasis by inhibiting SREBP and activating LXR. Moreover, oxysterols are widely involved in post-transcriptional regulation of cholesterol homeostasis by changing enzyme stability and/or activity (e.g., promoting HMGCR degradation, affecting the activity of several cholesterol biosynthetic enzymes, *etc.*) (Luu et al., 2016).

## 3 Reprogrammed cholesterol metabolism in tumor cells

**FIGURE 1:**
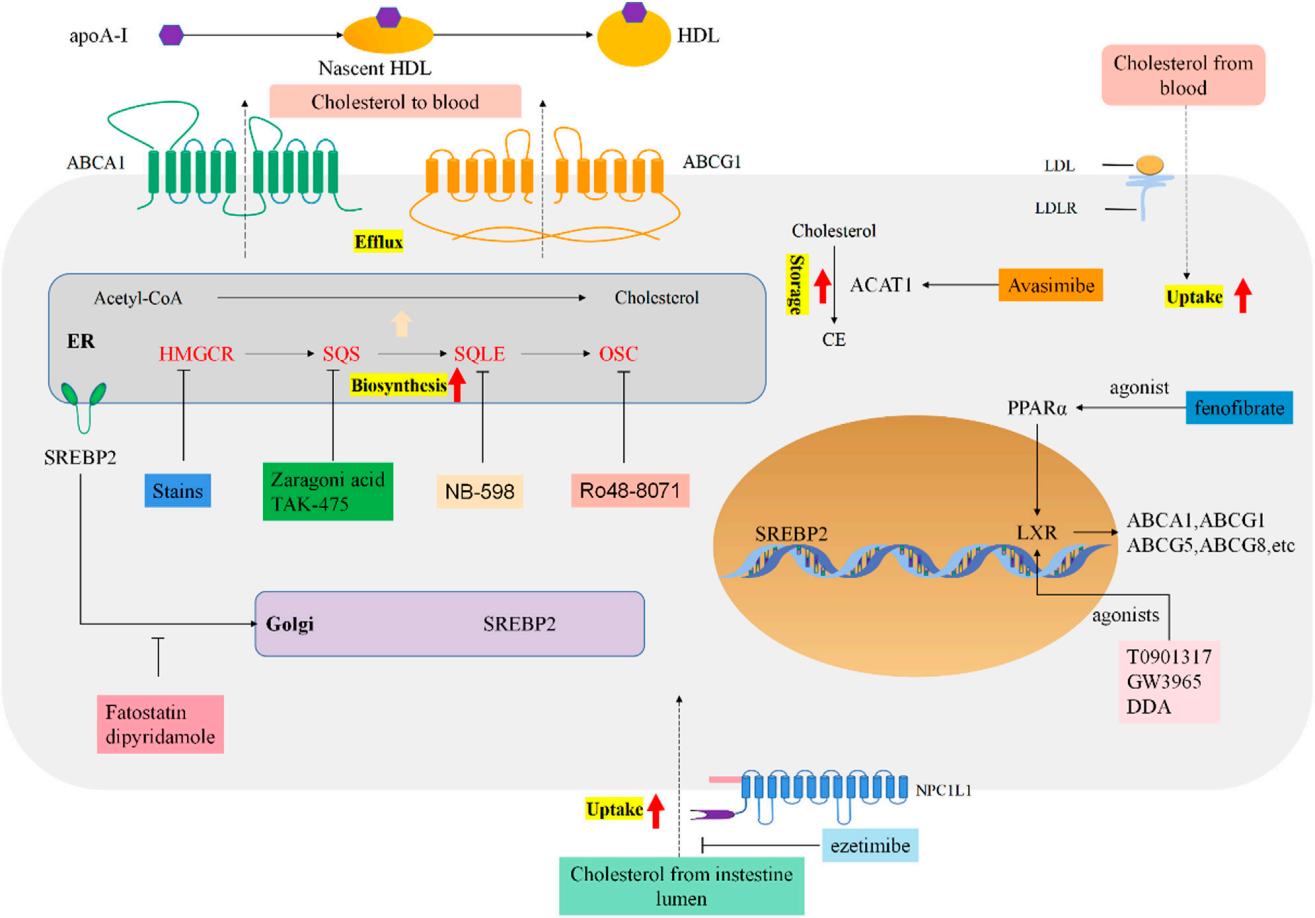
Reprogrammed cholesterol metabolism and indicated drug targets in tumor cells. Cholesterol metabolism and homeostasis regulation in cancer cells. Boxes of different colors indicate targeted therapeutic drugs and drug targets for each stage of cholesterol metabolism.

Cholesterol is generally beneficial for cancer growth and development, it promotes migration and invasion, inhibits apoptosis through activating oncogenic signaling pathways (Figure 1).

### 3.1 Cholesterol biosynthesis is enhanced in tumor cells

Tumor cells require excess cholesterol and intermediates of the cholesterol biosynthesis pathway to maintain cell proliferation, possibly related to the substantial cholesterol require for membrane synthesis (Cruz et al., 2013). Increased endogenous cholesterol synthesis and high cholesterol exposure both favor cancer progression (Kopecka et al., 2020b). Interestingly, intracellular cholesterol levels cause more cancer burden than systemic serum cholesterol, suggesting that abnormalities in cholesterol biosynthesis are strongly associated with tumorigenesis (Sorrentino et al., 2014; Kuzu et al., 2016).

Several enzymes such as SREBP2, HMGCR, SQS, OSC, and SQLE which are involved in cholesterol synthesis are significantly upregulated in liver cancer mouse model (Liang et al., 2018). SREBP2 and its downstream targets, including mevalonate-pathway enzymes, are significantly upregulated in glioblastoma (Lewis et al., 2015). HMGCR is overexpressed in prostate cancer, gastric cancer and colon cancer (Giacomini et al., 2021). Squalene synthase (SQS) is enhanced in lung cancer patients, induces cholesterol biosynthesis, which in turn maintains the enrichment of tumor necrosis factor receptor 1 (TNFR1) in lipid rafts to promote lung cancer metastasis (Yang et al., 2014). Inhibition of SQS reduces the levels of lipid raft-associated cholesterol, inhibits prostate cancer cell proliferation, and induces apoptotic (Brusselmans et al., 2007). The level of squalene cycloxidase (SQLE) is enhanced in breast cancer, lung cancer and colorectal cancer, and promotes cancer cell migration and invasion, which may be related to regulating the sterol components of lipid rafts as well (Giacomini et al., 2021). In metastatic mouse models of colorectal and pancreatic cancer, lanosterol synthase (LSS) promotes tumor neovascularization and metastasis (Maione et al., 2015). Oxide squalene cyclase (OSC) inhibitors hinder endothelial cell migration and promote apoptosis, which inhibits tumor angiogenesis and dissemination to the distance (Liang et al., 2014). In addition, enhanced expression of cholesterol synthesis genes is associated with poor survival in sarcoma, acute myeloid leukemia and melanoma patients, but in lower grade glioma it was associated with good survival (Kuzu et al., 2016). The latest research has revealed that activated cholesterol biosynthesis programs promotes triple-negative breast cancer progression (Cai et al., 2019) and increased cholesterol synthesis is associated with poor patient prognosis (Ehmsen et al., 2019).

Mechanistically, cholesterol biosynthesis has complex links with the signaling pathways and factors that regulate tumors. Several oncogenic signals such as PI3K/AKT/mTOR, RTK/RAS, and TP53 have been shown to modulate cholesterol synthesis in cancer cells (Kuzu et al., 2016). For example, constitutive activation of PI3K/AKT signaling increases intracellular cholesterol levels through SREBP-1 activation, resulting in *de novo* cholesterol biosynthesis and LDL receptor (LDLR) expression, thereby enhancing exogenous cholesterol import in prostate cancer (Guo et al., 2011). On the other hand, cholesterol biosynthesis also has a critical role in maintaining cancer stem cells by activating signaling pathways of sonic hedgehog, Notch and receptor tyrosine kinases (Kim, 2019). Thus, targeting the cholesterol generation and mevalonate pathway represents a promising choice for tumor therapy.

### 3.2 Cholesterol uptake is enhanced in tumor cells

Increasing cholesterol uptake appears to be more efficient strategy compared to *de novo* cholesterol synthesis for cancer cells. It is reported that NPC1L1 promotes colon carcinogenesis by inducing cholesterol absorption and increasing plasma cholesterol levels (He et al., 2015). NPC1L1 knockdown reduces colitis-associated tumorigenesis, which may be associated with downregulation of β-catenin, p-c-Jun and p-ERK (He et al., 2015). One of the extracellular loops of NPC1L1 is the binding site of ezetimibe, thus providing support for targeted cholesterol uptake (Weinglass et al., 2008). Besides, it has been found that some anaplastic large cell lymphoma cells are completely dependent on cholesterol uptake to acquire cholesterol, due to the absence of SQLE. These cancer cells actively upregulate LDLR, which takes up exogenous cholesterol as an alternative strategy to support proliferation (Garcia-Bermudez et al., 2019). Indeed, LDLRs levels are increased in glioblastoma, leukemia, pancreatic and lung cancers (Huang et al., 2016; Gallagher et al., 2017) and LDLRs promotes epithelial-to-mesenchymal transition (EMT), increases the secretion of metalloproteinase MMP-9 and activates Wnt/β-catenin signaling pathway (Campion et al., 2020). However, the level of LDLR is decreased in human advanced prostate cancer. The roles of hypercholesterolemia in tumors are still controversial: elevated serum cholesterol level is positively correlated with the recurrence rate of prostate cancer (Allott et al., 2014). But it is also reported that high serum cholesterol levels increased the anti-tumor functions of natural killer cells and reduced the growth of liver tumors in mice (Pelton et al., 2014). Collectively, while cholesterol uptake is one of the sources for cancer cells to obtain cholesterol, how cancer cells coordinate the balance between cholesterol biosynthesis and uptake and whether it is altered with tumor progression remains to be further elucidated.

### 3.3 Cholesterol efflux is dysregulated in tumor cells

Deficiency of ABCA1, a main receptor mediating cholesterol efflux, increases mitochondrial cholesterol, inhibits release of mitochondrial cell death-promoting molecules, and thus facilitates cancer cell survival (Smith and Land, 2012; Kuzu et al., 2016). It has been demonstrated that ABCA1 can promote cell metastasis by regulating cholesterol levels, and patients with high ABCA1 expression had shorter times to metastasis in breast cancer (Aguirre-Portoles et al., 2018). PPARα and PPARγ activation promotes LXR-mediated ABCA1 expression, and PPARα blocks cholesterol biosynthesis by inhibiting sterol regulatory element binding protein 2 (SREBP-2) activity (Grabacka and Reiss, 2008). Thus, targeting PPARα appears to be an effective strategy to regulate cholesterol content. Indeed, the antitumor effect of fenofibrate (an agonist of PPARα) has been demonstrated (Giacomini et al., 2021).

### 3.4 Cholesterol esterification is enhanced in tumor cells

As mentioned above, cells are able to avoid excessive cholesterol accumulation through the cholesterol esterification pathway. Usually, cholesterol esterification reduces the amount of intracellular free cholesterol, protects tumor cells from their toxic effects, and reduces the amount of free cholesterol that can maintain SREBP-induced cholesterol biosynthesis and uptake (Chang et al., 2006). However, it is also reported that reducing cholesterol esterification was able to inhibit the growth and invasion of hepatoma carcinoma cells in a mouse xenograft model (Geng et al., 2016), suggesting that the function of cholesterol esterification depends on tumor types. Cholesteryl esters (CE), a common signature in cancer, is usually stored in lipid droplets that serve as a reservoir for neutral lipids such as triacylglycerols. The accumulation of CE can be converted by tumor cells into cholesterol utilization, as demonstrated by high expression of ACAT1 and cholesterol ester metabolizing enzyme lysosomal acid lipase (LAL) in tumor tissues. In fact, the accumulation of CE promotes proliferation and invasive capacity of breast cancer, and promotes the occurrence and metastatic potential of glioblastoma, prostate and pancreatic cancer (de Gonzalo-Calvo et al., 2015; Petan et al., 2018). CE accumulation is driven by loss of PTEN and consequent activation of PI3K/AKT/mTOR pathway that induces the expression of SREBP and LDLR, thereby promoting ACAT1-mediated cholesterol storage in lipid droplets (Yue et al., 2014). In glioblastomas, inhibition of ACAT1 inhibits adipogenesis and tumor growth (Geng et al., 2016). Consistently, ACAT1 overexpression was confirmed in many cancers, including hepatocellular carcinoma, castration-resistant prostate cancer, and pancreatic cancer (Giacomini et al., 2021). Therefore, targeted enhanced cholesterol esterification seems to be a promising therapeutic strategy. In fact, it has been shown that targeting ACAT1 has an anticancer potential (Yue et al., 2014).

### 3.5 Abnormal regulation of cholesterol homeostasis in tumor cells

As mentioned above, SREBP2 and LXR are essential for maintaining cholesterol homeostasis. SREBP promotes cancer cell growth, migration, and colony generation in esophageal squamous cell carcinoma (Zhong et al., 2019). SREBP and its downstream genes are significantly upregulated and promote cell survival and tumor growth in the hypoxic and nutrient-restricted tumor microenvironment (Lewis et al., 2015). SREBP2 has also been shown to bind to mutant p53 and activate the expression of the mevalonate pathway in breast cancer cells (Freed-Pastor et al., 2012). Moreover, it is proved that RORγ (a nuclear receptor) promotes the recruitment of SREBP2, and activates the cholesterol biosynthesis (Cai et al., 2019). Thus, the SREBP and RORγ can serve as good targets for tumor therapy. In addition to SREBP, LXR is also an important driver of carcinogenesis. LXR inverse agonists and LXR agonists were shown to inhibit the proliferation and colony formation, and induce apoptosis in clear cell renal cell carcinoma (ccRCC) cells, but had no cytotoxic effect on normal renal tubular epithelial cells. Therefore, LXR may be a safe therapeutic target for ccRCC (Wu et al., 2019).

### 3.6 Oxysterols have multifunctional role in cancer cells

Oxysterols are involved in various cancers (Kuzu et al., 2016). Side-chain oxidation of cholesterol generates 22-hydrocholesterol (22-HC), 24-hydroxycholesterol (24-HC), 25-hydroxycholesterol (25-HC) and 27-hydroxycholesterol (27-HC), and oxidation occurring on the backbone generates 7α/β-hydroxycholesterol (7α-HC/7β-HC), 7-ketocholesterol (7-KC) and 5, 6α/β-epoxycholesterol (5, 6α -EC/5, 6β-EC). 22-HC is a high-affinity LXR ligand that induces ABCA1 expression, leading to cellular cholesterol efflux. 25-HC is a side-chain oxysterol that inhibits cholesterol biosynthesis by inhibiting SREBP (Riscal et al., 2019). Certain oxysterols have anticancer effects. In Jurkat T-cell lymphoma cells, 24-HC induces apoptosis through a mechanism involving 24-HC esters and lipid droplet accumulation (Yamanaka et al., 2014). 22-HC, 24-HC, 7α-HC/7β-HC and 5, 6α -EC/5, and 6β-EC all act as agonists of LXR to inhibit proliferation in breast cancer, ovarian cancer and prostate cancer through inducing G1 cell cycle arrest or apoptosis (Lin et al., 2013; Riscal et al., 2019; de Medina et al., 2021). Thus, oxysterols with cytotoxic activity may be potential therapeutic agents for cancer. However, 27-HC acts as an estrogen receptor (ER) agonist in breast cancer, which stimulates tumor growth and metastasis in multiple breast cancer models (McDonnell et al., 2014). A recent study demonstrated that chronic exposure of cancer cells to 27-HC, which likely models the situation in patients with hypercholesterolemia/dyslipidemia, resulted in the emergence of cells exhibiting increased tumorigenic and metastatic capacity (Liu et al., 2021). Intriguingly, the metabolites of 5, 6-epoxycholesterol (5, 6-EC) have opposing properties in breast cancer oncogenesis. In normal breast tissue, the metabolite dendrogenin A (DDA) displays tumour-suppressive properties. Yet in breast cancer, 5, 6-EC is metabolized to oncosterone (6-oxo-cholestan-3, 6-diol, cholestan-3, 6-diol-6-one, and OCDO), acting as an oncometabolite and tumor promoter in breast cancer. Therefore, blocking oncosterone biosynthesis or neutralizing oncosterone receptors may be a new pharmacological target for the treatment of breast cancer (de Medina et al., 2021).

Besides the tumor cells, oxysterols can also influence the tumor microenvironment. Immune cells expressing “generic” oxysterol receptors, such as LXR, and specific receptors in immune cells, such as G protein-coupled receptor 183 (GPR183), can recognize different oxysterols (Willinger, 2019). Baek et al. (2017) demonstrated that 27-HC increases the number and activity of polymorphonuclear neutrophils (PMN) and γδT cells, and reduces the cytotoxic CD8^+^ T cell population. In addition, oxysterol promotes tumor growth by inhibiting dendritic cell (DC) migration to lymphoid and by promoting the recruitment of protumor neutrophils in the tumor microenvironment (Raccosta et al., 2013).

## 4 Targeting cholesterol in tumor therapy

### 4.1 Targeting cholesterol biosynthesis

#### 4.1.1 Targeting HMGCR

As cholesterol metabolism has important functions in cancer progression, targeting cholesterol metabolism has been shown to be a viable antitumor strategy (Table 1). As previously described, HMGCR is one of the rate-limiting enzymes for the cholesterol-producing mevalonate pathway, so targeting HMGCR may be a good strategy for tumor therapy (Nielsen et al., 2012; Gu et al., 2019; Di Bello et al., 2020). Statins are the most common pharmacological inhibitors of HMGCR. Numerous epidemiological analyses suggest statins can reduce the incidence of certain tumors, but these conclusions are not consistent (Kuzu et al., 2016). One study suggests an association between statin and a slight reduction in cancer-related mortality for 13 different cancer types (Nielsen et al., 2012). However, there are also many epidemiological studies suggest no association between statin and cancer (Kuzu et al., 2016). Statins can enhance the effects of chemotherapeutic agents such as cisplatin, anthracyclines, paclitaxel, 5-fluorouracil, etoposide and malfaran (Osmak, 2012). The efficacy of reducing side effects and drug resistance has also been proved (Terzi et al., 2019; Feng et al., 2020). Currently, the efficacy of statins has been carried out in both basic studies and clinical trials to evaluate monotherapy and therapies in combination with other chemotherapeutic agents.

**TABLE 1:** Anti-cancer therapies that target cholesterol metabolism.

	Therapeutic class		Mechanism	Cancer type	References
Targeting cholesterol biosynthesis	Stains	Simvastatin Atorvastatin Lovastatin Pravastatin Rosuvastatin Fluvastatin Pitavastatin	Competitive inhibitors of HMGCR	Colorectal, Prostate, Breast, Lung cancer, multiple myeloma, melanoma and other cancers	Nielsen et al. (2012), Osmak. (2012), Fatehi Hassanabad. (2019), Gu et al. (2019), Terzi et al. (2019), Chen Y H et al. (2020), Di Bello et al. (2020), Feng et al. (2020), Lubtow et al. (2020), Okubo et al. (2020)
	Zaragonic acids		Inhibitor of squalene synthase	RMA lymphoma and Lewis lung carcinoma models	Brusselmans et al. (2007), Lanterna et al. (2016)
	TAK-475		Inhibitor of squalene synthase (FDFT1)	Pancreatic ductal adenocarcinoma model	Biancur et al. (2021)
	NB-598		Inhibitor of squalene epoxidase	SCLC lines	Mahoney et al. (2019)
	R048-8071		Inhibitor of OSC	HCT116 CRC, HPAF-II pancreatic adenocarcinoma models and breast cancer lines	Liang et al. (2014), Maione et al. (2015)
Targeting cholesterol uptake	ezetimibe		Selective block of NPC1L1	Breast cancer	Pelton et al. (2014)
Targeting cholesterol efflux	fenofibrate		PPARα agonists	Leukemia, Lymphoma, Multiple Myeloma, endometrial cancer, prostate cancer, breast cancer, oral cancer, pancreatic cancerand and other cancers	Luo et al. (2019), Sun et al. (2019), You et al. (2019), Chen L et al. (2020), Di Bello et al. (2020)
Targeting cholesterol storage	Avasimibe		Inhibitor of ACAT1	Human PC3 prostate cancer, MIA-PaCa2 pancreatic cancer, A549 lung cancer, and HCT116 colon cancer lines	Pal et al. (2013), Lee et al. (2015), Lee et al. (2018), Li et al. (2018)
Targeting cholesterol regulation	Fatostatin		specific inhibitor of SREBP	Prostate cancer, ER-positive breast cancer	Li et al. (2014), Li et al. (2015), Gao et al. (2018), Liu et al. (2020), Yao et al. (2020)
	dipyridamole		inhibit the cleavage of SREBP2	multiple myeloma	Pandyra et al. (2014)
	T0901317		LXR agonists	Breast, lung, prostate cancer and Leukemia	Pommier et al. (2010), El Roz et al. (2012), Flaveny et al. (2015), Villa et al. (2016), Tavazoie et al. (2018), Lou et al. (2019), Brendolan and Russo. (2022)
	GW3965			Leukemia	
	DDA (Dendrogenin A)		LXR partial agonist	Leukemia	
	RGX-104		LXRβ agonist	Advanced solid tumors and lymphomas	
	SR9243		LXR inverse agonist	Colorectal, lung, prostate cancer models	

While inhibiting cholesterol biosynthesis, statins also inhibit the synthesis of multiple other metabolites. By blocking the MVP pathway, statins halt isoprenoid synthesis, such as GGPP and FPP for GTPase-proteins essential for cancer cells (Takai et al., 2001), which explains the pharmacological effects of statins in antitumor effects (Takai et al., 2001; Buhaescu and Izzedine, 2007; Kidera et al., 2010). Moreover, the antitumor effects of statins may also be related to non-MVP-mediated mechanisms (Okubo et al., 2020).

Since statins have been approved for the treatment of hypercholesterolemia and are one of the most widely used pharmaceutical agents in the world. Thus, their repositioning in the field of oncology is translated more easily and quickly to the clinic. From the first clinical trial of lovastatin combined with cytarabine started in 2001, how statins work and benefit in cancers therapy has been widely evaluated over these 2 decades. When “statins | cancer” are taken as the search term, 223 clinical trials have been found on ClinicalTrial.gov, including 54 phase I studies, 101 phase II studies, 24 phase III studies and 12 phase IV studies from 2005 to 2023. Based on the types of diseases, studies for clinical oncology treatment-related trials were included in the analysis (Table 2).

**TABLE 2:** Clinical trials of statins in cancer.

Drug	Cancer type	Condition	Phase	Combination strategy	References
**Simvastatin**	Breast Cancer		II	Anastrozole	Bao et al. (2012)
		with dyslipidemia	II	-	
		prevention	II	-	Lazzeroni et al. (2012)
		ER-positive/metastatic	II	Fulvestrant, Metformin	
		metastatic	II	HER2-targeted therapy	
	Prostate Cancer		II	Metformin	
			I	Ezetimibe	Wang et al. (2022)
	Colorectal Cancer	metastatic	II	FOLFIRI (irinotecan, 5-FU, leucovorin)	
		advanced/metastatic	II	Cetuximab/Panitumumab/Bevacizumab	
	Lung cancer SCLC		II	Irinotecan/Albumin Paclitaxel/Irinotecan, Cisplatin	
	NSCLC		II	gefitinib	Han J Y et al. (2011)
**Atorvastatin**	Breast cancer		II	Letrozole	
		triple negative	II	Zoledronate	
		early stage	III	-	
	Prostate Cancer		II	Celecoxib	
		prevent recurrence	II	-	Jeong et al. (2021)
	Glioblastoma multiforme		II	Temozolomide	Altwairgi et al. (2021)
	Hepatocellular Carcinoma (HCC)	prevent recurrence	II	Metformin	
		advanced	II	Sorafenib	
	Colorectal Cancer	prevention	II	-	
	pancreatic cancer	metastatic	I	Ezetimibe, Evolocumab	
**Lovastatin**	Ovarian cancer	refractory/relapsed	II	Paclitaxel	
	Breast Cancer	prevention	II	-	Vinayak et al. (2013)
	Melanoma		II	Interferon alfa-2b	
		precancerous lesions	II	-	Linden et al. (2014)
**Pravastatin**	HCC	advanced	II/III	Sorafenib	Jouve et al. (2019), Riaño et al. (2020), Blanc et al. (2021)
	Leukemia	prevent recurrence	II	Cytarabine/Idarubicin	Advani et al. (2014), Shadman et al. (2015)
		relapsed/refractory	I/II	Cyclosporine, Mitoxantrone Hydrochloride, Etoposide	Chen et al. (2013)
	Lung Cancer SCLC		III	Etoposide, Cisplatin/Carboplatin	Seckl et al. (2017)
**Rosuvastatin**	Endometrial Carcinoma	Stage I	II	Megestrol Acetate	
	Colorectal Cancer	prevent recurrence/advanced	II/III	-	
**Fluvastatin**	Breast Cancer		II	-	Garwood et al. (2010)
	Prostate Cancer		II	-	Longo et al. (2020)
**Pitavastatin**	Breast Cancer		II/III	-	

Of the 15 studies with results released, 5 suggested positive anti-tumor outcomes, including simvastatin: 1 (1/2), pravastatin: 2 (2/7), fluvastatin: 2 (2/2) in NSCLC, breast cancer, prostate cancer, leukemia and HCC.

A phase II study has been carried out to evaluate the efficacy and safety of gefitinib plus simvastatin in patients with advanced non-small cell lung cancer (NSCLC). The result pointed out that there is no superiority of GS (gefitinib plus simvastatin) to G (gefitinib only) was demonstrated in the unselected NSCLC population. But GS showed a higher response rate (RR) and longer progression-free survival (PFS) compared with G alone in patients with wild-type EGFR non-adenocarcinomas (Han J.-Y. et al., 2011). Several studies of simvastatin combination treatment in small cell lung cancer (SCLC) are ongoing. Another study tested the effects of simvastatin on the pharmacokinetics of anastrozole, a potent non-steroidal aromatase inhibitor (AI) that holds promise for breast cancer prevention, on patients with hormone receptor-positive breast cancer suggested that simvastatin is not likely to compromise the activity of anastrozole (Bao et al., 2012). While, a study of simvastatin in patients at higher risk of developing a hormone non-responsive (ER-) breast cancer was carried out in 2011 (NCT01500577). This study included 150 women with a history of estrogen receptor negative ductal intraepithelial neoplasia or lobular intraepithelial neoplasia or atypical hyperplasia, or unaffected subjects carrying a mutation of BRCA1 or with a probability of mutation >10% (according to BRCAPRO) (Lazzeroni et al., 2012) to evaluate the chemoprevention activity of simvastatin compared with nimesulide. And the result of this trial has not yet been released.

A study of breast cancer patients with a 3–6 weeks fluvastatin treatment before surgery suggested measurable biologic changes by reducing tumor proliferation and increasing apoptotic activity in high-grade, stage 0/1 breast cancer (Garwood et al., 2010) (NCT00416403). A phase II study in prostate cancer patients shows that short-term (4–12 weeks) fluvastatin treatment at a cholesterol-lowering dose before radical prostatectomy can increase the percentage of apoptotic prostate cancer cells in the tumor relative to baseline (Longo et al., 2020) (NCT01992042).

A positive result for high dose pravastatin combined with cytarabine and idarubicin in relapsed AML patients’ therapy was reported in 2014 (Advani et al., 2014) (NCT00840177). The recurrence rate has decreased from 75% to 5.5% after the combined treatment, which shows the efficacy of this combined therapy. While another study had been ceased due to the combined drugs did not meet the predefined efficacy criteria for success (Shadman et al., 2015) (NCT01831232).

As for HCC, there are three phase II studies aim to bring out the efficacy of sorafenib combined with statins to select better arms for further clinical trials in patients with advanced hepatocellular carcinoma (HCC), as sorafenib is the preferred drug in the palliative treatment [NCT01418729 (Riaño et al., 2020), NCT01357486 (Blanc et al., 2021), NCT01075555 (Jouve et al., 2019)]. All these three studies showed that adding pravastatin to sorafenib did not improve overall survival (OS) in patients with advanced HCC. However, one of the studies suggested the combination of sorafenib and pravastatin prolonging the time to progression (TTP) of patients with advanced HCC (Blanc et al., 2021).

Despite of the positive outcome of multiple types of Statins drugs in clinical trials mentioned above, there are still some unsatisfactory results. The included studies related to atorvastatin and lovastatin did not suggest a positive outcome. For example, atorvastatin has been evaluated in the prevention of the recurrence of prostate cancer, which has shown that there was no association with a lower risk of disease recurrence compared with placebo (Jeong et al., 2021). While glioblastoma patients treated with atorvastatin in combination with radiotherapy and temozolomide did not show an improvement in progression-free survival (Altwairgi et al., 2021). In addition, evaluation of lovastatin as a prevention drug for its use in the treatment of women at increased risk of breast cancer demonstrated no significant biomarker modulation (NCT00285857) (Vinayak et al., 2013). Besides, there is a study of lovastatin in melanoma, which did not show beneficial changes of lovastatin for precancerous lesions (Linden et al., 2014) (NCT00462280). Some studies had been terminated due to the toxicity of drug combination (Chen et al., 2013) (NCT01342887). There are also trials being recruited or underway, and for those without positive results, longer observation periods and larger sample sizes are needed to determine the therapeutic effects of statins on various types of tumors. Besides, for trials with poor outcomes, distinguishing more subgroups, such as gene polymorphism and smoking (Han J.-Y. et al., 2011; Han J. Y. et al., 2011). May lead to meaningful conclusions. Moreover, the safety of statins still needs to be given enough attention when used in combination with chemotherapeutic drugs, and individual differences in drug use for cancer patients also need to be considered.

#### 4.1.2 Targeting squalene synthase

Squalene protects cancer cells from ferroptotic cell death, providing a growth advantage under conditions of oxidative stress produced by high proliferative rates and in tumor xenografts (Garcia-Bermudez et al., 2019). It has been experimentally demonstrated that Zaragozionic acid, a pharmacological inhibitor of Squalene synthase (SQS), can lead to growth arrest and induction of cytotoxicity in prostate cancer cells (Brusselmans et al., 2007). In addition, using TAK-475, a potent inhibitor of squalene synthase (Fdft1), researcher evaluated the efficacy and tolerability of TAK-475 in a mouse transplant model of pancreatic ductal adenocarcinoma (PDA) and showed significantly reduced tumor growth (Biancur et al., 2021).

#### 4.1.3 Targeting SQLE

A recent study showed that increased squalene production due to the loss of squalene epoxidase (SQLE) in cholesterol nutrient-deficient cells prevents oxidative cell death (Garcia-Bermudez et al., 2019). Mahoney et al. (2019) demonstrated that small cell lung cancer (SCLC) lines display sensitivity to NB-598, a known inhibitor of squalene epoxidase (SQLE). In addition, terbinafine (TB) is an antifungal agent that inhibits squalene epoxidase and has been shown to inhibit tumor growth and angiogenesis (Chien et al., 2012), by the mechanism that TB suppresses *in vitro* and *in vivo* proliferation of various tumor cells, including oral, colon and liver cancer *via* inhibiting DNA synthesis and activating apoptosis, which is related to the p53-dependent signaling pathway (Lee et al., 2003).

#### 4.1.4 Targeting OSC

Oxide squalene cyclase (OSC) is the enzyme that catalyzes the conversion of a 2,3-monoepoxy squalene to a lanosterol. Since lanosterol is a precursor to cholesterol, inhibition of OSC leads to reduced cholesterol synthesis, experimental evidence has demonstrated anti-antitumor effects of OSC inhibitors in human glioblastoma and brain-derived endothelial cells and enhanced antitumor effects in combination with statins (Staedler et al., 2012). Ro 48–8071, an OSC inhibitor, shows anti-tumor effect (Maione et al., 2015), and more importantly, it synergizes with 5-fluorouracil, thus eliciting an enhanced anti-tumor outcome.

### 4.2 Targeting cholesterol uptake

Administration of a low-cholesterol diet or ezetimibe (an inhibitor of NPC1L1) reduces tumor growth by reducing cholesterol levels (Pelton et al., 2014). In addition, it has been demonstrated that the use of leelamine (a lysosomotropic compound, intercellular cholesterol transport inhibitor) suppresses autophagic flux and induces cholesterol accumulation in lysosomal/endosomal cell compartments, disrupts lysosomal cell compartments, and induces cancer cell death (Kuzu et al., 2014). High dietary cholesterol can bypass the need to enhance endogenous cholesterol synthesis, thus accelerate the development of liver cancer. Moreover, major cholesterol metabolites, such as 27HC, 25HC, 22HC, and 6-oxocholsterol-3β, 5α-diol, can promote tumorigenesis (Nelson, 2018; Riscal et al., 2019). Furthermore, to maintain systemic cholesterol homeostasis and reduce ATP depletion of *de novo* cholesterol biosynthesis, some cancer cells alter mevalonate pathway enzyme expression and deregulate cholesterol influx/efflux genes, such as VLDLR, LDLR, SR-B1 and ABCA1, which in turn may lead to cancer cell resistance to statins (Riscal et al., 2019). Therefore, combining a low cholesterol diet or the use of cholesterol absorption inhibitors (such as ezetimibe) with anticancer drugs may be a promising strategy for clinical treatment of tumors.

Vytorin^®^, a combination drug which contains ezetimibe (10 mg) and simvastatin (40 mg), was used in an early phase I study to determine whether cholesterol-lowering therapy could slow the growth of prostate cancer (NCT02534376). The result shows that Ki-67 staining decreased in normal prostate tissue and low-grade prostate cancers and there was no significant change in Ki-67 staining in high-grade prostate cancers. This suggests that cholesterol-lowering therapy may decrease growth in both benign prostate that produces voiding symptoms in older men and low-grade prostate cancer (Wang et al., 2022). An ongoing Phase I trial will evaluate a PCSK9-inhibitors (evolocumab) in combination with atorvastatin and ezetimibe in patients with metastatic pancreatic cancer undergoing standard chemotherapy (NCT04862260).

### 4.3 Targeting cholesterol efflux

Synthetic bette agonists (including fenofibrate) have been used as lipid-lowering therapeutic agents. In addition to the lipid-lowering effects, drugs targeting PPARα also have therapeutic effects in cancer. In fact, Luo et al. (2019) found that intestinal depletion of PPARα promotes colon carcinogenesis by increasing DNMT1-mediated p21 methylation and PRMT6-mediated methylation of p27. While using fenofibrate activated PPAR and inhibited colon carcinogenesis (Luo et al., 2019). It has been shown that fenofibrate inhibition of cell proliferation simultaneously suppresses the expression of key enzymes in fatty acid metabolism and induces human hepatoma Hep3B cells apoptosis (You et al., 2019). In addition, it has been demonstrated that fenofibrate has anti-cancer effects in endometrial cancer, prostate cancer, triple negative breast cancer, oral cancer and pancreatic cancer (Sun et al., 2019; Chen L et al., 2020). Chen L et al. (2020) demonstrated that fenofibrate could induce mitochondrial reprogramming through activation of the AMPK pathway and inhibition of the HK2 pathway, inhibiting gastric cancer cell proliferation and promoting apoptotic through the PPARα pathway. Therefore, targeting PPARα may be an effective cancer treatment and has been tested in clinical trials. When “fenofibrate/bezafibrate | cancer” are taken as the search term, 18 clinical trials have been found on ClinicalTrial.gov, including a phase I study, 6 phase II studies, 5 phase III studies from 2006 to 2023 (Table 3).

**TABLE 3:** Clinical trials of fibrate in cancer.

Drug	Cancer type	Phase	Combined drug	
Fenofibrate	Central Nervous System Tumor, Pediatric	II	Celecoxib Cyclophosphamide Etoposide Thalidomide	Robison et al. (2014)
	Leukemia			
	Lymphoma			
	Neuroblastoma			
	Sarcoma			
	Multiple Myeloma	II	-	
Bezafibrate	Myelodysplastic Syndromes (MDS)	II	Sodium Valproate Medroxyprogesterone	

A phase II trial of a multi-agent oral antiangiogenic regimen in children with recurrent or progressive cancer had been carried out in 2006 (NCT00357500). “5-drug” regimen, including celecoxib, cyclophosphamide, etoposide, thalidomide, and fenofibrate, was evaluated in patients with eight diseases. Of 97 patients, 24 patients completed 27 weeks of therapy without progression. As a result, the combination of drugs had shown clinical benefits in patients with low-grade glioma and ependymoma (Robison et al., 2014). And the mitochondrial inhibitory function of fenofibrate was tested in a clinical phase II study in patients with multiple myeloma (NCT01965834).

Of the three included studies, one trial on fenofibrate had results and suggested a positive clinical oncology effect. For now, there are fewer clinical trials of fibrates for oncology treatment. More clinical studies can be conducted to confirm the effectiveness of fibrates in the future.

### 4.4 Targeting cholesterol storage

High expression of ACAT1 is related to cell proliferation rates, tumor formation and metastasis, and cell resistance (Giacomini et al., 2021). Indeed, treatment of breast cancer cells with ACAT-1 inhibitors resulted in reduced cell proliferation and migration and reduced tumor growth through regulation of cholesterol metabolism (Antalis et al., 2010; Shim et al., 2018). Avasimin, a systemically injectable nanoformulation containing the ACAT-1 inhibitor avasimibe has been developed, which has been used in clinical trials for the treatment of atherosclerosis and shows good human safety (Pal et al., 2013; Lee et al., 2015). The formulation was tested in different human cancer cell lines showing that avasimin reduces lipid droplet accumulation in prostate cancer cells and reduces cellular activity in a variety of tumor cell lines (Lee et al., 2015). ACAT-1 was overexpressed in MIA PaCa-2 human pancreatic cancer cells compared to normal cells, and treatment of cells with avasimibe or knockdown of the ACAT-1 gene results in a block of cholesterol esterification, and a decrease in cell invasion and migration. This may be because ACAT-1 inhibition impairs Wnt/β-catenin signaling, thereby overcoming cancer cell metastasis (Lee et al., 2018). The combination of gemcitabine and avasimbe showed synergistic effects *in vitro* and may overcome gemcitabine resistance for pancreatic ductal adenocarcinoma treatment (Li et al., 2018).

### 4.5 Targeting cholesterol regulation

#### 4.5.1 Targeting SREBP

Fatostatin, a specific inhibitor binds the SREBP-cleavage activating protein (SCAP) to block cholesterol biosynthesis, is able to inhibit tumor growth *in vivo* in a mouse prostate cancer experiment (Li et al., 2014). In endometrial cancer, Fatostatin reduces cancer cell viability and tumor growth in xenografted mice and improves their survival rate (Gao et al., 2018). It has also been demonstrated that Fatostatin inhibit the growth and proliferation of human endometrial cancer cells, alter its cell cycle and induce apoptotic (Yao et al., 2020). Furthermore, Fatostatin can induce ER degradation by polyubiquitination of K48 junctions, a key mechanism for tamoxifen to inhibit PI3K-AKT-mTOR signaling in breast cancer, and has a synergistic effect with tamoxifen in reducing cell proliferation *in vitro and in vivo* tumor growth in breast cancer, indicating that Fatostatin may have promising clinical use for ER-positive breast cancer patients (Liu et al., 2020). In addition, the combination of Fatostatin and docetaxel resulted in greater proliferation inhibition and apoptosis induction compared with single agent treatment in PCa cells *in vitro* an,d *in vivo*, especially those with mutant p53s (Li et al., 2015). Of note, dipyridamole was also shown to inhibit the cleavage of SREBP2. The statin–dipyridamole combination was synergistic and induced apoptosis in multiple myeloma and AML cell lines and primary patient samples, whereas normal peripheral blood mononuclear cells were not affected (Pandyra et al., 2014).

#### 4.5.2 Targeting RORγ

The RORγ was identified as an important driver of the cholesterol biosynthesis program. RORγ inhibition would counteract the statin-induced SREBP2-dependent feedback regulation and reduce the tumor cholesterol biosynthesis rate without affecting the host cholesterol homeostasis (Cai et al., 2019). Indeed, ROR inhibitors cooperate with statins to kill TNBC (triple-negative breast cancer) cells, and in addition, ROR-selective antagonists are very effective manifested by leading tumor regression and blocking metastasis in multiple TNBC models (Cai et al., 2019).

#### 4.5.3 Targeting LXR

LXR can be activated by endogenous ligands, such as oxysterol or by agonists. In MCF-7 breast cancer cells, treatment with two LXR agonists (TO901317 and 22 (R) -hydroxycholesterol) can inhibit MCF-7 cells proliferation and induce their apoptosis (El Roz et al., 2012). In prostate cancer, the AKT survival pathway was downregulated by treatment with the LXR agonist T0901317, thereby inducing the apoptotic of LNCaP PCa cells in xenograft nude mice and cell cultures (Pommier et al., 2010). Furthermore, it has been demonstrated that the combination treatment of T0901317 and anticancer drug gefitinib exhibits synergistic effects in lung cancer models, inhibiting lung cancer migration and invasion *in vivo* and *in vitro*, which may be through inhibition of ERK/MAPK signaling pathway (Lou et al., 2019). In hematopoietic malignancies, the agonists of LXR (T0901317, GW3965 and DDA) can induce apoptosis or lethal autophagy in leukemic cells (Brendolan and Russo, 2022). The treatment of primary acute myeloid leukemia (AML) samples with dendrogenin A (DDA), a modulator of LXR, that is, a partial LXR agonist, induces lethal autophagy *in vitro* and *in vivo* (de Medina et al., 2021; Brendolan and Russo, 2022). Meanwhile, exogenous 27-Hydroxycholesterol induces apoptosis in leukemic cells (HL60, KG1α, and K562 cells) through the accumulation of reactive oxygen species (ROS) (Woo et al., 2022). In addition, because LXR is a transcription factor towards to different targets including genes associated with glycolysis and lipogenesis, targeting this receptor may be a promising approach for cancer therapy. Interestingly, a reverse agonist SR9243 was designed, and SR9243 inhibits LXR activation by enhancing LXR-corepressor recruitment (Flaveny et al., 2015). It has been demonstrated that SR9243 can induce apoptosis in leukemic cells. In contrast, as was previously described, the activation of LXR by different agonists has also been shown to reduce cancer cell survival by promoting cholesterol efflux, especially in glioblastoma (Villa et al., 2016).

Very recently, the latest trial was just posted on *Clinicaltrials* (ClinicalTrials.gov) on 23 January 2023 which is initiated in 2016 (ClinicalTrials.gov Identifier: NCT02922764). This is a phase I, dose escalation and expansion study of RGX-104, an oral small molecule targeting the LXR. By depleting both myeloid-derived suppressor cells (MDSCs) and tumor blood vessels, it exerts its anti-tumor activity (Tavazoie et al., 2018). This trial will evaluate single agents or combinations in patients with advanced solid tumors and lymphomas. Combinations include nivolumab, ipilimumab, docetaxel, or pembrolizumab plus carboplatin/pemetrexed. In the expansion stage, the study will provide further characterization of the safety, efficacy, PK, and pharmacodynamics. Immunological activity and biomarkers of LXR target activation will also be evaluated.

The statins, as well as ezetimibe and fibrates mentioned in the above clinical trials, are all approved in the blood cholesterol guideline, which demonstrate their safety and feasibility for oncology treatment (Grundy et al., 2019). In the last 3 years, there were 29 ongoing phase II or III clinical trials for oncology treatment with statins alone or in combination with other drugs, 13 of which were first posted in these 3 years. Other targeted drugs related to cholesterol metabolism are also gaining attention. These trials focus on the prevention of cholesterol metabolism-related drugs in patients at high risk for cancer, the treatment of further disease progression, and the prevention of recurrence in cancer patients, and are primarily focused on breast, prostate, small cell lung, intestinal and uterine cancers. Furthermore, other trials focusing on the prevention and treatment of side effects of chemotherapy and radiotherapy for tumors, such as heart failure, hearing loss (Fernandez et al., 2021), and metabolic syndrome, which are not selected for analysis but show the promise of this class of drugs in oncology treatment.

## 5 Conclusion and perspectives

Cholesterol is one of the important nutrients for normal physiological function, the latest Dietary Guidelines for Americans and Chinese removed the restriction for dietary cholesterol. However, we should think calmly about dietary cholesterol and health. Restricted dietary cholesterol intake in people at high risk of cardiovascular disease is recommended in many guidelines. In addition, dietary cholesterol is just one aspect of a healthy diet. Population health is closely related to the overall dietary pattern. We should not only pay attention to a separate aspect of the food, but also consider the interactive effects of multiple foods. Besides, Current dietary guidelines limit saturated fatty acids to 10% of total energy, and dietary cholesterol intake is generally not too high if people meet this requirement.

Cholesterol is normally linked to cardiovascular diseases. Recently, there has been extensive evidence demonstrating that cardiovascular disease and cancer are intertwined. Firstly, cardiovascular disease and cancer share several common risk factors, including diabetes, dyslipidemia, cachexia, and an impaired immune response. Secondly, Anticancer therapies can induce CVD *via* several mechanisms, including direct cardiotoxicity, effects on the vasculature, and perturbations to cardiovascular and immune homeostasis (Curigliano et al., 2012; Karlstaedt et al., 2022). Thirdly, patients with cardiovascular disease have higher cancer risk compared with individuals from the general population (a concept referred to as reverse cardio-oncology) (Aboumsallem et al., 2020; Karlstaedt et al., 2022; Koelwyn et al., 2022).

In this review, it is evident that cholesterol metabolism is critical for cancer progression and targeted drugs including statins and fibrates are widely used in clinical trials (Huang et al., 2020; Xu et al., 2020). However, there are still a number of outstanding questions in the field need to be further addressed. Firstly, in cholesterol metabolism targeted therapy, the maintenance of cholesterol homeostasis is more important than just lowers the level of cholesterol. Secondly, the accurate metabolic subtypes of cancers should be established for better applying metabolic therapy. Thirdly, it is not so clear that the effect of cholesterol metabolism on immune microenvironment which also plays the key roles upon tumor therapy. So far, the efficacy of targeted cholesterol metabolism therapy largely depends on cancer types and all targeted drugs are not used as first-line drugs but used in combination with other therapy. Besides directly targeting cholesterol metabolism, bile acid, the main product of cholesterol transformation, directly affects the intestinal microflora, and the microecology is closely related to the occurrence and prognosis of cancers. Therefore, we should also focus on the microecology of intestinal microflora while detecting cholesterol levels inside and outside tumor cells. Nevertheless, all these progressions from bench to bed make targeting cholesterol metabolism therapy a fascinating field to work in, and targeted therapy which is more effectively, safely, precisely and comprehensively should be further investigated.
